# Transient Phase-Driven Cyclic Deformation in Additively Manufactured 15-5 PH Steel

**DOI:** 10.3390/ma15030777

**Published:** 2022-01-20

**Authors:** Tu-Ngoc Lam, Yu-Hao Wu, Chia-Jou Liu, Hobyung Chae, Soo-Yeol Lee, Jayant Jain, Ke An, E-Wen Huang

**Affiliations:** 1 Department of Materials Science and Engineering, National Yang Ming Chiao Tung University, Hsinchu 30010, Taiwan; lamtungoc1310@gmail.com (T.-N.L.); paradise7139@gmail.com (Y.-H.W.); jasmine861025@gmail.com (C.-J.L.); 2 Department of Physics, College of Education, Can Tho University, Can Tho 900000, Vietnam; 3 Department of Materials Science and Engineering, Chungnam National University, Daejeon 34134, Korea; highteen5@cnu.ac.kr; 4 Department of Materials Science and Engineering, Indian Institute of Technology, New Delhi 110016, India; 5 Chemical and Engineering Materials Division, The Spallation Neutron Source, Oak Ridge National Laboratory, Oak Ridge, TN 37831, USA; kean@ornl.gov

**Keywords:** 15-5 PH stainless steel, in-situ neutron diffraction, selective laser melting, low-cycle fatigue, martensite transformation

## Abstract

The present work extends the examination of selective laser melting (SLM)-fabricated 15-5 PH steel with the 8%-transient-austenite-phase towards fully-reversed strain-controlled low-cycle fatigue (LCF) test. The cyclic-deformation response and microstructural evolution were investigated via in-situ neutron-diffraction measurements. The transient-austenite-phase rapidly transformed into the martensite phase in the initial cyclic-hardening stage, followed by an almost complete martensitic transformation in the cyclic-softening and steady stage. The compressive stress was much greater than the tensile stress at the same strain amplitude. The enhanced martensitic transformation associated with lower dislocation densities under compression predominantly governed such a striking tension-compression asymmetry in the SLM-built 15-5 PH.

## 1. Introduction

Selective laser melting (SLM), one of the most commonly used approaches in additive manufacturing (AM) technique, provides great abilities in fabricating layer by layer-built parts with complex geometry and customization, which significantly affects the anisotropy in mechanical properties with respect to the building directions [1,2,3,4,5]. The heterogeneous microstructure with the formation of defects in the SLM-manufactured alloys is inherently different from the homogeneous microstructure of the conventional cast and wrought alloys, causing unpredictable mechanical and fatigue properties of the SLM-fabricated components [2,6,7,8]. During the consecutive cycles of rapid heating and solidification under laser melting, the occurrence of defects, nucleation and growth, and phase transformation induces a metastable microstructure of the additive manufactured metals [2,9,10].

The imperfections such as pores are strongly governed by the welding environments, which significantly decreased the mechanical properties of the steel welded joints [11]. Garcias et al. compared the material properties fabricated via conventional manufacturing and AM methods in which porosity and lack of fusion in the additive manufactured steels were found to have a negative effect on their mechanical and fatigue responses [12]. Post processing may reduce the imperfections and improve the properties of additive manufactured alloys [12]. Tailoring the intricate AM process with plenty fabrication conditions associated with various post treatments is full of challenges but a requisite for great optimization of mechanical and fatigue performances of the additive manufactured alloys.

The AM process parameters enable the controllable microstructure of precipitation hardened stainless steels consisting of austenite and martensite phases and thus strongly govern their mechanical properties [13,14,15,16]. The retained austenite produced in the additive manufactured steels during AM process enabled the transformation-induced plasticity [17]. Investigations on the influence of non-equilibrium retained austenite γ-phase and the strain-induced martensitic transformation under loading in the additive manufactured 15-5 PH and 17-4 PH steels have been extensively reported [4,14,18,19,20,21,22,23,24,25]. Huang et al. examined the tensile properties of the 15-5 PH steel with a tunable fraction of metastable transient-austenite-phase, which can be successfully tailored via the SLM process. They demonstrated that a higher fraction of transient austenite γ-phase was beneficial to the tensile properties of the SLM-built 15-5 PH steel [4]. Specifically, the SLM-built 15-5 PH with the 18%-transient-austenite-phase revealed a more impressive strain hardening effect beyond yielding, while the specimen with the 8%-transient-austenite-phase disclosed similar deformation behavior as the as-quenched martensite steel [4]. Chae et al. demonstrated the principal strengthening mechanisms via various heat treatments in tailoring the mechanical properties of the additive manufactured 15-5 PH steels [26].

In the other alloy systems, deformation-induced phase development was found, such as in the cobalt-based superalloys during monotonic and cyclic deformation [27]. Meanwhile, although comprehensive studies have been devoted to deciphering the strain-induced phase transformation under monotonic tensile loading, the cyclic-induced phase transformation subjected to low-cycle-fatigue (LCF) deformation in the SLM-built 15-5 PH steel conducted by in-situ neutron diffraction is unclear and thus intriguing to examine.

We herein extend our earlier works [4,24,28] towards the microstructural evolution of α-matrix and transient γ-phase under cyclic continuous tension-compression loading. We explore the fully-reversed strain-controlled LCF deformation at a strain amplitude of ±1.0% in the SLM-built 15-5 PH with the 8%-transient-austenite-phase at room temperature. In-situ neutron-diffraction measurement was carried out to examine the cyclic-stress response during symmetric tension-compression cyclic loading by monitoring the microstructural evolutions [29], such as the dislocations [30], which are beneficial to elucidate the corresponding deformation mechanisms governing fatigue behavior of the SLM-built 15-5 PH. The possible factors governing the applied stress response upon cyclic loading were reported.

## 2. Materials and Methods

**Figure 1:**
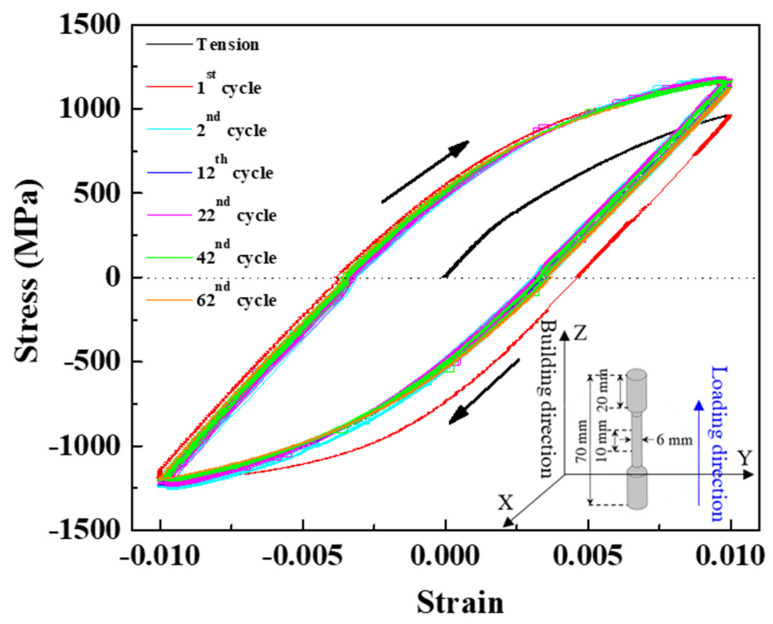
Stress-strain curves under LCF test in the SLM-built 15-5 PH steel. Schematic illustration of the vertically-built SLM 15-5 PH specimen is shown in the inset.

The additive manufactured 15-5 PH steel was fabricated from the PH1 powder via SLM. The chemical composition was composed of 14.35 wt% Cr, 4.03 wt% Ni, 2.71 wt% Cu, 0.01 wt% Mn, 0.99 wt% Si, 0.5 wt% Mo, 0.74 wt% Nb, <0.01 wt% C, and Fe balance. The laser power of 195 W, spot size of 70 μm, and scanning rate of 1200 mm/s were used. The vertically-built SLM 15-5 PH steel with the 8%-transient-austenite-phase used in this study was produced such that the building direction was parallel to the loading direction, shown in the inset of Figure 1. More details of the 8%-transient-austenite-phase specimen can be referred in our previous study [4].

In-situ neutron-diffraction measurements were carried out using the VULCAN engineering diffractometer at the Spallation Neutron Source (SNS) of the Oak Ridge National Laboratory (ORNL) [31,32,33,34]. The neutron-diffraction profiles were recorded simultaneously in both loading and transverse directions using two orthogonal detectors situated at ±90° from the incident neutron beam. The axial and transverse detectors collect the diffraction patterns from crystallographic orientations parallel and perpendicular to the applied loading direction, respectively. The LCF test was conducted with a maximum tensile strain of 1.0% and a maximum compressive strain −1.0% at a cyclic frequency of 1 Hz. The cylindrical dog-bone-shaped specimen used for LCF loading had a total length of 70 mm, a gauge length of 10 mm, and a diameter of 6 mm, shown in the inset of Figure 1. Neutron-diffraction profiles were recorded during LCF test in a strain-controlled mode at maximum tensile and compressive strains of 1.0% and −1.0% of each fatigue cycle until the specimen was fractured. The procedures for in-situ neutron-diffraction experiments and data analysis followed our earlier protocol [4]. The neutron-diffraction patterns were fitted using the general structure analysis system II (GSAS-II) [35] for phase characterization and profile analysis. Moreover, the development of dislocation density during LCF response for both α-matrix and transient γ-phase was determined from the peak-width evolution following Tomota’s method [36]. The evolution of phase fraction was obtained from the refined peak intensity as a function of fatigue cycles.

## 3. Results

### 3.1. Cyclic Stress-Strain Curves

Figure 1 presents the hysteresis loops at several selected fatigue cycles under LCF test at a strain amplitude of ±1.0% in the SLM-built 15-5 PH steel. The initial loading was in tension. In the 1st cycle, the stress value was 955 MPa at 1.0% strain. During unloading and compression, the plastic deformation started, and the stress value was 1158 MPa at −1.0% strain, respectively. In reloading to tension, the stress value increased to 1118 MPa at 1.0% strain, originating from the significant strain hardening from the 1st to 2nd cycles. In the 2nd cycle, during unloading to zero, plastic strain decreased but the stress value at −1.0% strain was higher compared to the 1st cycle. The softening and saturation stages then occurred up to the 62nd cycle at which the specimen was fractured.

### 3.2. Cyclic-Induced Martensitic Transformation

**Figure 2:**
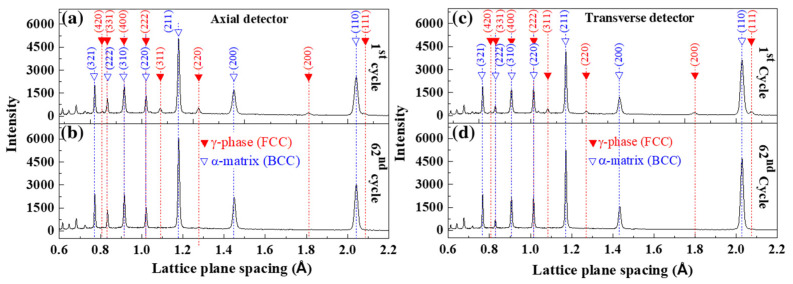
In-situ neutron-diffraction profiles at the maximum tensile strain of 1.0% in the axial loading direction at the (**a**) 1st cycle and (**b**) 62nd cycle. Those in the transverse direction at the (**c**) 1st cycle and (**d**) 62nd cycle in the SLM-built 15-5 PH steel.

Figure 2 depicts the evolution of in-situ neutron-diffraction patterns at 1.0% strain at the 1st and 62nd cycles in both axial loading and transverse directions. The diffraction profiles of body-centered cubic (BCC) α-matrix and face-centered cubic (FCC) γ-phase austenite assigned for each (h k l) were illustrated as the blue and red arrows, respectively. The intensity of (1 1 0) peak of α-matrix in the transverse direction (Figure 2c) was greater by 30% than that in the axial loading direction in the 1st cycle (Figure 2a). Upon cycling, the intensity of α-matrix peaks (ferrite and martensite) significantly increased while that of γ-phase peaks dramatically decreased and almost disappeared at the 62nd cycle in both axial loading (Figure 2b) and transverse directions (Figure 2d). The neutron results indicated the cyclic-induced martensitic transformation in the SLM-built 15-5 PH during continuous tension-compression loading.

### 3.3. Tension-Compression Asymmetry Behavior

**Figure 3:**
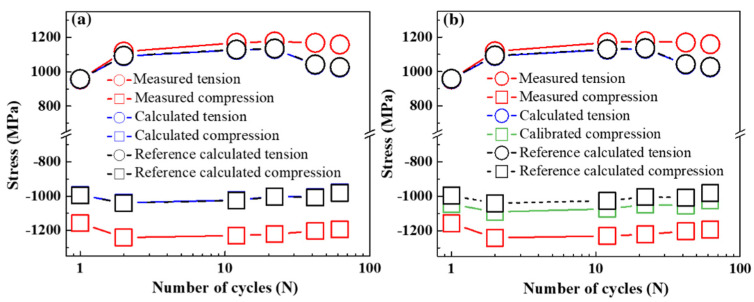
(**a**) The measured stress, calculated stress, and reference calculated stress at the maximum tensile and compressive deformations as a function of fatigue cycles. (**b**) The calculated compressive stress in (**a**) was replaced by the calibrated compressive stress.

Figure 3a describes the cyclic-stress amplitudes at the tensile strain of 1.0% (red circles ○) and compressive strain of −1.0% (red squares □) as a function of fatigue cycles from 1 to 62 under LCF response in the SLM-built 15-5 PH steel. Under tensile deformation, the specimen experienced an initial cyclic hardening in which the applied stress significantly increased from 955 MPa at the 1st cycle to 1177 MPa at the 22nd cycle, followed by a slightly cyclic-softening and steady behavior up to the 62nd cycle. Meanwhile, under compressive deformation, there was an increase of cyclic stress from 1158 MPa to 1241 MPa at the 2nd cycle where the cyclic-hardening/softening transition occurred, followed by a more obvious cyclic-softening and steady behavior until the 62nd cycle. Moreover, the compressive stress was much higher than the tensile stress at the same strain amplitude, suggesting an asymmetric response in tensile-compressive deformation in the SLM-built 15-5 PH steel. Such a remarkable tension-compression asymmetry behavior is one of the typical characteristics of the additive manufactured alloys [37,38].

Since the applied stresses vary differently under tension and compression at the same strain amplitude, clarifying the relationship between the macroscopic applied stress and microscopic lattice strain is necessary. We followed Wang et al.’s [39] and Young et al.’s [40] methods to estimate the effective phase stresses in understanding the role of α-matrix and γ-phase under applied stresses. We calculated the average von Mises equivalent stress for each phase [41] on the assumption that two orthogonal transverse strain components ($\sigma_{11}=\sigma_{22}$) are equal for cylindrical dog-bone sample. We applied the generalized Hooke’s law shown as below.

(1) $$\sigma_{11}^{\alpha }=\sigma_{22}^{\alpha }=\frac{E}{\left(1+\upsilon \right)\left(1-2\upsilon \right)}\left\{\left(1-\upsilon \right)\varepsilon_{11}^{\alpha }+\upsilon \left(\varepsilon_{22}^{\alpha }+\varepsilon_{33}^{\alpha }\right)\right\},$$

(2) $$\sigma_{33}^{\alpha }=\frac{E}{\left(1+\upsilon \right)\left(1-2\upsilon \right)}\left\{\left(1-\upsilon \right)\varepsilon_{33}^{\alpha }+\upsilon \left(\varepsilon_{22}^{\alpha }+\varepsilon_{11}^{\alpha }\right)\right\},$$

where *E* is Young’s modulus, υ is Poisson’s ratio, $\sigma_{33}$ and $\varepsilon_{33}$ are the stress and strain components along the axial loading direction, respectively, $\sigma_{11}$ and $\sigma_{22}$ are the stress components in the transverse direction, $\varepsilon_{11}$ and $\varepsilon_{22}$ are the strain components in the transverse direction, and α is the BCC α-matrix. The same calculation is applied for the FCC γ-phase by substituting γ for α.

The calculated stress ($\sigma_{ij}^{calculated}$) can be estimated as follows

(3) $$\sigma_{ij}^{calculated}=\sigma_{ij}^{\alpha }\left(1-f\right)+\sigma_{ij}^{\gamma }f,$$

where *f* is the volume fraction of the FCC γ-phase, $\sigma_{ij}^{\alpha }$ and $\sigma_{ij}^{\gamma }$ are the stress components of the BCC α-matrix and FCC γ-phase, respectively.

Figure 3a presents the calculated tensile and compressive stresses obtained from the generalized Hooke’s law shown in the blue circles (○) and squares (□), respectively, while the reference calculated tensile and compressive stresses using the reference Young’s modulus [42] were depicted in the black circles (○) and squares (□), respectively. It can be seen from Figure 3a that the calculated stresses were almost the same as the reference calculated stresses under both tensile and compressive deformations. Furthermore, the measured stress was much closer to the reference calculated stress under tension than under compression.

### 3.4. Effect of Porous Structure on the Tension-Compression Asymmetry Behavior

The outstanding tension-compression asymmetry behavior may be governed by various reasonable factors such as porous structure, phase transformation, and dislocation density. The pores, microvoids, or inclusions commonly exist in the additive manufactured steels due to thermal gradients or unmelted powder during the additive manufacturing process [37,43,44,45]. Thus, we first assumed that porous structure is one of the possible causes resulting in the tension-compression asymmetry behavior. To correlate the mechanical properties of porous materials with their relative densities, we recalled the Ashby and Gibson model (1997) as follows [46]:

(4) $$\sigma_{y}=\sigma_{y0}\times \rho_{rel}^{3/2},$$

(5) $$E_{eff}=E_{0}\times \rho_{rel}^{2},$$

where $\sigma_{Y}$ and $E_{eff}$ are the yield strength and effective modulus of the porous material, respectively; $\sigma_{y0}$ and $E_{0}$ are the yield strength and elastic modulus of the bulk material, respectively; and $\rho_{rel}$ is the relative density of the porous material.

We herein assumed that when the specimen experiences tensile deformation, the internal pores are stretched and the volume becomes larger. However, the internal pores are squeezed or even disappeared as the specimen undergoes compressive deformation. The deformation is presumably highly influenced by large internal porosity in tension. Therefore, the parameters of the porous material were expressed as the case of tension while the parameters of the bulk material were represented as the case of compression. Based on the measured Young’s moduli obtained from the slopes of lattice strain versus engineering stress curves under tension and compression, the relative densities of α-matrix and γ-phase calculated from Equation (5) were 0.970 and 0.794, respectively. The stress components of α-matrix and γ-phase of the porous material under compression were accordingly adjusted by Equation (4) instead of Equation (2). The calibrated compressive stress was then calculated by Equation (3), which was shown in the green squares (□) in Figure 3b. Compared with the calculated compressive stress, the calibrated compressive stress was closer to the measured compressive stress. Despite taking the porous structure into consideration, the calibrated compressive stress was still inconsistent with the measured compressive stress, suggesting other factors governing the tension-compression asymmetry behavior besides the porous structure.

### 3.5. Effect of Microscopic Change on the Tension-Compression Asymmetry Behavior

**Figure 4:**
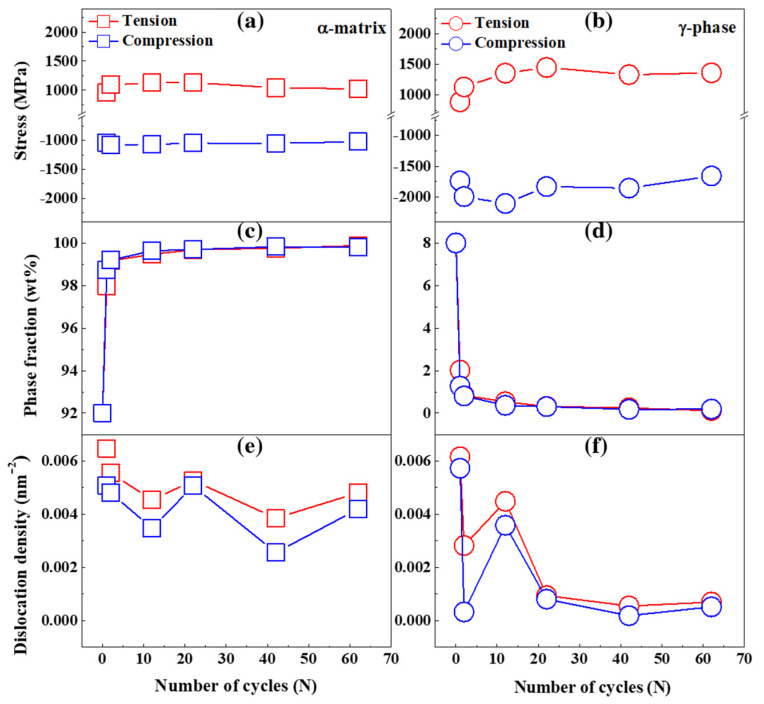
The microscopically individual stresses of the (**a**) α-matrix and (**b**) γ-phase; the phase fraction of the (**c**) α-matrix and (**d**) γ-phase; the dislocation density of the (**e**) α-matrix and (**f**) γ-phase as a function of fatigue cycles.

Since the two phases of α-matrix and γ-phase co-existed and there was martensitic transformation in the SLM-built 15-5 PH steel during cyclic loading, exploring the contribution of each phase to the macroscopically measured stresses under both tension and compression is necessary. Figure 4a,b present the microscopically individual stresses of α-matrix and γ-phase as a function of fatigue cycles, respectively. The individual stress of α-matrix disclosed an obvious symmetry under tension and compression; however, the individual stress of γ-phase revealed an evident tension-compression asymmetry behavior upon cycling. The macroscopic tension-compression asymmetry was probably originated from the major contribution of γ-phase instead of α-matrix. It should be noted that the phase fraction should be taken into account when calculating the macroscopic stress of the specimen by Equation (3). Particularly, the phase fraction of γ-phase austenite (8 wt%) was much lower than that of α-phase ferrite (92 wt%) before the fatigue test. Moreover, the γ-phase austenite rapidly transformed to the martensite phase up to the 2nd cycle, followed by a nearly complete martensitic transformation upon further cycling, as seen in the phase fraction evolution of α-matrix and γ-phase in Figure 4c,d, respectively. Since there is a very small fraction of γ-phase austenite upon cycling, its effect on the macroscopic stress should be trivial. Therefore, the tension-compression asymmetry of γ-phase had a negligible influence on the macroscopic tension-compression asymmetry behavior in the SLM-built 15-5 PH.

Accompanying with the martensitic transformation, examination of microstructural evolution of the individual phases during cyclic loading is indispensable to understand the tension-compression asymmetry in the SLM-built 15-5 PH steel. Figure 4e,f show the dynamic evolution of dislocation densities of α-matrix and γ-phase as a function of fatigue cycles, respectively. The evolution of dislocations under tension and compression disclosed similar trends in both α-matrix and γ-phase, however, the dislocations exhibited lower densities in compression than in tension for both α-matrix and γ-phase, implying the different contribution of dislocation activities to the tension-compression asymmetry in the SLM-built 15-5 PH specimen.

## 4. Discussion

Since the pores are usually formed in the fabricated alloys during SLM process and they strongly affect their mechanical properties, we further calculated the porosity of the SLM-built 15-5 PH by the Equation (6) [47].

(6) $$P(\%)=\left(1-\frac{\rho_{measured}}{\rho_{theoretical}}\right)*100,$$

where $\rho_{measured}$ is the measured density of the SLM-built 15-5 PH specimen and $\rho_{theoretical}$ is the theoretical density of the material.

The measured density of the SLM-built 15-5 PH measured by the Archimedes method was 7.759 g/cm^3^, which was similar to the density of the SLM-built 17-4PH steel with similar manufacturing parameters measured by Hengfeng Gu et al. [48]. The porosity of the SLM-built 15-5 PH specimen was determined to be 0.53%, which is insufficient to have a major impact on the tension-compression asymmetry behavior [49]. The results inferred that the porosity had relatively minor contributions to the tension-compression asymmetry behavior in the SLM-built 15-5 PH specimen.

The phase fraction evolution of both α-matrix and γ-phase was similar under tension and compression in Figure 4c,d, however, there was a noticeable difference in the amount of martensitic transformation between tension and compression. A slightly higher fraction of the cyclic-induced martensite phase was visible under compression than under tension in the cyclic-hardening region, while no evident discrepancy of martensitic transformation was seen in the cyclic-softening and steady stage. Since the martensite phase is harder than the other phases, the more pronounced fraction of cyclic-induced martensite phase under compression significantly contributes to the higher cyclic compressive stress at the same strain amplitude and thus the more hardening behavior under compression.

In Figure 4e,f, although the dislocation densities of both α-matrix and γ-phase decreased with increasing fatigue cycles, the dislocation densities of γ-phase dramatically reduced upon cycling. Such a drastic degradation of dislocation density of γ-phase leads to the cyclic-softening region [50]. Furthermore, the dislocation densities of both α-matrix and γ-phase were lower under compression rather than under tension. The decreased dislocation densities under the fatigue test were also observed under the monotonic tensile test in the SLM-built 15-5 PH, which was assigned to the coalescence of dislocations during martensitic transformation [4]. Lower dislocation densities under compression promote the nucleation and growth of the martensite phase [50], which is beneficial to the higher cyclic stress under compression.

## 5. Conclusions

We examined the cyclic-stress response under strain-controlled LCF test in the SLM-built 15-5 PH steel with 8%-transient-austenite-phase and identified the possible underlying factors. The SLM-built 15-5 PH specimen was fractured at the 62nd cycle upon cycling loading at a strain amplitude of ±1.0%. The compressive stresses of 1158 MPa and 1241 MPa were higher than the tensile stresses of 955 MPa and 1118 MPa at the 1st and 2nd cycles, respectively. The effect of inherent pores and porosity on the SLM-built 15-5 PH plays a secondary role in higher cyclic compressive stress. Such a hardening response under compression was mainly ascribed to the primary martensitic transformation coupled with lower dislocation densities. Understanding the principal microstructural changes governing the applied stress amplitude upon cycling is helpful for the design of fatigue-resistant additive manufactured alloys.

## Data Availability

The data presented in this study are available on request from the corresponding author.

## Nomenclature

Selective laser melting	SLM
Low-cycle fatigue	LCF
Additive manufacturing	AM
Spallation Neutron Source	SNS
Oak Ridge National Laboratory	ORNL
General structure analysis system II	GSAS-II
Body-centered cubic	BCC
Face-centered cubic	FCC
○	Measured tensile stress
□	Measured compressive stress
○	Calculated tensile stress
□	Calculated compressive stress
○	Reference calculated tensile stress
□	Reference calculated compressive stress
□	Calibrated compressive stress
E	Young’s modulus
υ	Poisson’s ratio
$\sigma_{33}$	Stress along the axial loading direction
$\varepsilon_{33}$	Strain along the axial loading direction
$\sigma_{11}$	Stress components in the transverse direction
$\sigma_{22}$	Stress components in the transverse direction
$\varepsilon_{11}$	Strain components in the transverse direction
$\varepsilon_{22}$	Strain components in the transverse direction
α	BCC α-matrix
γ	FCC γ-phase
$\sigma_{ij}^{calculated}$	Calculated stress
f	Volume fraction of FCC γ-phase
$\sigma_{ij}^{\alpha }$	Stress components of BCC α-matrix
$\sigma_{ij}^{\gamma }$	Stress components of FCC γ-phase
$\sigma_{Y}$	Yield strength of porous material
$E_{eff}$	Effective modulus of porous material
$\sigma_{y0}$	Yield strength of bulk material
$E_{0}$	Elastic modulus of bulk material
$\rho_{rel}$	Relative density of porous material
P(%)	Porosity
$\rho_{measured}$	Measured density
$\rho_{theoretical}$	Theoretical density
